# NAVIGATING THE CHALLENGES OF RETIREMENT TRANSITIONS FOR COUPLES IN ARAB SOCIETY

**DOI:** 10.1093/geroni/igad104.3488

**Published:** 2023-12-21

**Authors:** Reem Nashef-Hamuda

**Affiliations:** Haifa University, Qalansawa, Hefa, Israel

## Abstract

Retirement marks the beginning of the third age and is considered one of the most significant transitions in the later stages of life In the aging process, individuals and couples must adapt to many changes that affect their lifestyle and marital relationships. This study investigates post-retirement couples’ dynamics in Arab society, focusing on Relational Turbulence Theory (RTT) and its correlation with marital satisfaction and intimacy. Data from 106 retired couples (average male age = 70.3, female age = 65.1) reveal RTT’s relevance in explaining relationship uncertainty and interdependence during transitions. Using Confirmatory Factor Analysis, psychometric properties are validated, and gender equivalence is established. Actor-Partner Interdependence Modeling demonstrates that self-uncertainty, partner uncertainty, relationship uncertainty, interference, and facilitation significantly predict marital quality and intimacy. Partners’ impact is crucial. emphasizing how a partner’s experiences influence marital outcomes. These findings contribute to a deeper understanding of the complexities of post-retirement relationships in Arab society. The main theoretical contribution of the current study will be linking gerontology and couple dynamics in the context of the Arab society in Israel, which is an ethnic minority group and a traditional society in transition (modernization). the Practical implications of this study are helping professionals understand the factors that affect the quality of a couple’s relationship after retirement. This understanding will help professionals to provide adequate interventions for couples pre and postretirement, furthermore, the study’s findings will contribute to developing retirement planning interventions and culturally sensitive intervention programs for couples from the Arab society in Israel postretirement.

